# PGPointNovo: an efficient neural network-based tool for parallel *de novo* peptide sequencing

**DOI:** 10.1093/bioadv/vbad057

**Published:** 2023-04-25

**Authors:** Xiaofang Xu, Chunde Yang, Qiang He, Kunxian Shu, Yuan Xinpu, Zhiguang Chen, Yunping Zhu, Tao Chen

**Affiliations:** The School of Computer Science and Technology, Chongqing University of Posts and Telecommunications, Chongqing 400065, China; The School of Computer Science and Technology, Chongqing University of Posts and Telecommunications, Chongqing 400065, China; School of Software and Electrical Engineering, Swinburne University of Technology, Melbourne, Victoria 3122, Australia; Chongqing Key Laboratory on Big Data for Bio Intelligence, Chongqing University of Posts and Telecommunications, Chongqing 400065, China; Department of General Surgery, First Medical Center, Chinese PLA General Hospital, Beijing, China; School of Computer Science and Engineering, Sun Yat-Sen University, Guangzhou 26469, China; State Key Laboratory of Proteomics, Beijing Proteome Research Center, National Center for Protein Sciences (Beijing), Beijing Institute of Lifeomics, Beijing 102206, China; State Key Laboratory of Proteomics, Beijing Proteome Research Center, National Center for Protein Sciences (Beijing), Beijing Institute of Lifeomics, Beijing 102206, China

## Abstract

**Summary:**

*De novo* peptide sequencing for tandem mass spectrometry data is not only a key technology for novel peptide identification, but also a precedent task for many downstream tasks, such as vaccine and antibody studies. In recent years, neural network models for *de novo* peptide sequencing have manifested a remarkable ability to accommodate various data sources and outperformed conventional peptide identification tools. However, the excellent model is computationally expensive, taking up to 1 week to process about 400 000 spectrums. This article presents PGPointNovo, a novel neural network-based tool for parallel *de novo* peptide sequencing. PGPointNovo uses data parallelization technology to accelerate training and inference and optimizes the training obstacles caused by large batch sizes. The results of extensive experiments conducted on multiple datasets of different sizes demonstrate that compared with PointNovo the excellent neural network-based *de novo* peptide sequencing tool, PGPointNovo, accelerates *de novo* peptide sequencing by up to 7.35× without precision or recall compromises.

**Availability and implementation:**

The source code and the parameter settings are available at https://github.com/shallFun4Learning/PGPointNovo.

**Supplementary information:**

Supplementary data are available at *Bioinformatics Advances* online.

## 1 Introduction

In tandem mass spectrometry (MS/MS)-based proteomics, *de novo* peptide sequencing is a key technology for identifying novel peptides and unsequenced organisms (Li *et al.*, 2017). Without relying on sequence databases, *de novo* peptide sequencing can directly derive a peptide sequence from an MS/MS spectrum and is a significant tool for studying novel proteoforms caused by post-translational modifications or mutations (Vitorino *et al.*, 2020). In recent years, neural network models for *de novo* peptide sequencing have been developed to adapt to various sources of spectra data. These models have achieved superior performance. DeepNovo (Tran *et al.*, 2017) was proposed as the first tool that leverages convolutional neural network and long short-term memory in *de novo* peptide sequencing. Taking a step forward, PointNovo (Qiao *et al.*, 2021) was proposed to accommodate various data sources and data resolutions. It is one of the finest neural network-based tool for *de novo* peptide sequencing.

However, the volumes of MS data have been increasing explosively, caused by the rapid development of high-throughput sequencing and mass spectrometry instruments (Chen *et al.*, 2022). For example, there has been a rapid increase in the number and size of mass spectra data generated from 2017 to 2022 recorded by iProX (Supplementary Fig. S1). In iProX, 1294 datasets exceed 10 GB, 187 datasets exceed 100 GB, and 11 datasets exceed 1 TB (Supplementary Fig. S2). Such a large amount of available data is both an opportunity and a challenge. In practice, due to the influence of memory and computing power, PointNovo is difficult to use large-scale data sets for training on a single graphics processing unit (GPU). For example, PointNovo takes up to 1 week to process about 400 000 spectrums. This has become a major obstacle to the further and wider applications of neural network-based *de novo* peptide sequencing. There is an urgent need for efficiency improvement in processing large-scale spectra datasets. PyTorch now supports distributed data parallelization, which makes it possible to accelerate neural network-based peptide identification through data parallelization. But this is no easy task; data parallelization usually means that the model may need to compensate for the reduced steps with a larger learning rate, which may lead to convergence disobedience expectations.

In this article, we present PGPointNovo, a neural network-based *de novo* peptide sequencing tool that can process large-scale MS data in parallel. It parallelizes the peptide sequencing process of PointNovo—an excellent open-source tool for *de novo* peptide sequencing based on a specifically trained neural network. Briefly speaking, this is achieved by data parallelism—partitioning a large spectra dataset across multiple GPUs to be processed in parallel. To our best knowledge, PGPointNovo is the first open-source tool capable of parallel neural network-based *de novo* peptide sequencing. Extensive experiments conducted on multiple datasets of different sizes running on 8 GPUs demonstrate that, compared with PointNovo, PGPointNovo accelerates *de novo* peptide sequencing by up to 7.35× without precision or recall concession.

## 2 Methods

PGPointNovo parallelizes *de novo* peptide sequencing by partitioning a spectra dataset into multiple data partitions so that these data partitions can be processed by multiple GPUs—the basic processing units for neural network models—individually and simultaneously. The workflow of PGPointNovo consists of a training phase and a testing phase, as shown in Supplementary Figure S3. In the training phase, PGPointNovo creates a model, initializes it and deploys it on GPUs for training.

PGPointNovo implements data parallelization based on PointNovo. It is consistent with PointNovo in the data preprocessing stage. In the new data distribution stage, there are two principles: (i) Within an epoch, data sampling will not overlap, and all data will eventually be used up. (ii) In different epochs, different random seeds are used to ensure the diversity of data sampling. PGPointNovo will register autograd hooks to synchronize gradients during construction. After GPUs complete a training step, an all-reduce operation is performed to synchronize gradients, and all GPUs will be updated with the same gradient. It is described in more detail in Supplementary Figure S4. This operation ensures the model consistency across the GPUs—the same model can be obtained from any of these GPUs. In the testing phase, since no gradients are generated, it is sufficient to replace all-reduce with results collection. Through experiments, we discovered an interesting issue with parallel *de novo* peptide sequencing based on a neural network—an increase in batch size may cause a decrease in the model’s precision or ability to generalize, which is consistent with previous findings (Jia *et al.*, 2018; Kurth *et al.*, 2017). To address this issue, PGPointNovo employs an advanced optimizer named Ranger (Wright, 2019) as an option to reap the benefits of big batch size.

Based on the characteristics of *de novo* peptide sequencing, we have employed a series of cutting-edge techniques to further improve the performance of PGPointNovo: (i) employing Rectified Adam (Liu *et al.*, 2020), which explicitly rectifies the variance of the adaptive learning rate based on derivations to reduce the instability in early training; (ii) employing Lookahead (Zhang *et al.*, 2019), which maintain two sets of weights to lessen the need for extensive hyperparameter tuning; (iii) employing Gradient Centralization (Yong *et al.*, 2020), which introduces a new constraint on weight vector to strengthen generalization performance.

## 3 Results

We ran PGPointNovo on a single node of the super-computer in National Center for Protein Sciences (Beijing). This node has two 2.6 GHz Intel Xeon processors, eight Tesla V100 16GB GPUs and 256 GB RAM. To evaluate the performance of PGPointNovo through a fair and reproducible experimental comparison, we conduct experiments on the four datasets (Supplementary Table S2) originally used to evaluate PointNovo (Qiao *et al.*, 2021). For a fair comparison, PGPointNovo has used the same parameters as PointNovo, and more information on parameter is provided in Supplementary Table S1.

***Acceleration:*** Supplementary Table S3 compares PGPointNovo’s time-consuming against PointNovo. We can see that PGPointNovo achieves a 1.91x–7.35x training speedup and a 1.97x–7.11x inference speedup against PointNovo. The times taken for PGPointNovo and PointNovo to process a dataset both increase with the dataset size. PointNovo takes 86.55 h to process the PXD010559 dataset while PGPointNovo takes only 11.83 h with the support of 8 GPUs. The results indicate that PGPointNovo has a superior ability to process large datasets.

***Performance:*** As shown in Supplementary Table S4, we provide the performance of PGPointNovo on three benchmark datasets. While maintaining a linear acceleration ratio, PGPointNovo does not sacrifice identification precision or recall and shows even better performance than PointNovo on the larger PXD010559 dataset. In response, we conducted rigorous ablation tests based on a larger dataset and evaluated the generalizability of the model (as shown in Supplementary Table S5). The results demonstrate the generalizability of PGPointNovo and the availability of the Ranger.

## 4 Conclusion

With the rapid increase in mass spectrum data, it is a grand challenge to sequence a huge volume of data efficiently. This article presents PGPointNovo, an efficient neural network-based tool for parallel *de novo* peptide sequencing with data parallelism. Extensive experiments conducted on multiple datasets of different sizes demonstrate that PGPointNovo achieves profound speedups against the excellent approach without sacrificing precision, recall and generalizability.

## Supplementary Material

vbad057_Supplementary_Data
