# Supplementary material for: PGPointNovo: an efficient neural network-based tool for parallel de novo peptide sequencing
Source: Bioinform Adv. 2023 Apr 25;3(1):vbad057. doi: 10.1093/bioadv/vbad057 (PMC10148685; doi:10.1093/bioadv/vbad057)
Supplement: vbad057_Supplementary_Data [file vbad057_supplementary_data.pdf]

PGPointNovo: an efficient neural network-based tool for parallel *de novo* peptide sequencing

Xiaofang Xu[1], Chunde Yang[1], Qiang He[3], Kunxian Shu[5], Zhiguang Chen[4,*], Yunping Zhu[2,*] and Tao Chen[2,*]

[1]The School of Computer Science and Technology, Chongqing University of Posts and Telecommunications, Chongqing 400065, China, [2]State Key Laboratory of Proteomics, Beijing Proteome Research Center, National Center for Protein Sciences (Beijing), Beijing Institute of Lifeomics, Beijing 102206, China, [3]School of Software and Electrical Engineering, Swinburne University of Technology, Melbourne, Victoria 3122, Australia, [4]School of Computer Science and Engineering, Sun Yat-Sen University, Guangzhou 26469, China, [5]College of Bioinformation, Chongqing University of Posts and Telecommunications, Chongqing 400065, China

Supplementary Material

In recent years, various *de novo* sequencing tools based on neural networks have achieved remarkable performance. However, these tools incur high computational costs and often take a long time to process large-scale spectra datasets. It limits the *de novo* tool to play its due role. To overcome this obstacle, this paper presents PGPointNovo, a novel tool that parallelizes neural network based *de novo* sequencing across multiple GPUs based on data parallelism. Compared with PointNovo, the excellent neural network based *de novo* peptide sequencing tool, it achieves profound speedup gains without accuracy compromises.

Content

Part1. Description and illustration of figures, charts and methods in Efficient PointNovo 3

Part2. Availability, Installation and Usage of Efficient PGPointNovo 12

Part3. Conclusion and forecast of the research 13

REFERENCES 13

Part1. Description and illustration of figures, charts and methods in PGPointNovo

Supplementary Table S1. Key Parameters of PGPointNovo

	Parameters	PointNovo	PGPointNovo
Identical Parts	epochs	20	20
	beam size	5	5
	batch size	16	16
Different parts	learning rate	0.001	0.001*GPUs
	steps per validation	300	300/GPUs
	optimizer	Adam	Adam or Ranger

To ensure that the experiment is fair, we need to state the key parameters explicitly. Linearly expanding the learning rate is a common practice in parallelization. This is due to the fact that parallelization disguises an increase in batch size and a decrease in steps, so the model needs to converge by a sufficiently large step size. Similarly, PointNovo performs validation every 300 steps by default, and the reduction of steps in PGPointNovo requires a corresponding reduction in the number of steps between each validation.Finally, we include the Ranger as an option to cope with the potential loss of precision/recall caused by parallelisation.

Apart from this, all biological parameters remain the same. This includes modifications, upper mass limits, maximum lengths, etc. More details are presented in Supplementary Table S2 and `config.py`.

Supplementary Table S2. Information on Datasets

**Dataset**	ABRF	PXD008844	PXD010559	The merged dataset			
**Data Name**	ABRF	PXD008844	PXD010559	PXD008808	PXD011246	PXD012645	PXD012979
**Species**	Homo sapiens (Human)	Mus musculus (Mouse)	Plasmodium berghei ANKA; Anopheles stephensi (Asian malaria mosquito); Bos taurus (Bovine); Homo sapiens (Human)	Tursiops truncatus (Atlantic bottle-nosed dolphin) (Delphinus truncatus)	Homo sapiens (Human)	Homo sapiens (Human)	Mus musculus (Mouse)
**Modifications (Used by PointNovo)**	fixed: C(Carbamidomethylation); variable: M(Oxidation),N(Deamidation),Q(Deamidation)	fixed: C(Carbamidomethylation); variable: M(Oxidation)	fixed: C(Carbamidomethylation); variable: M(Oxidation), N(Deamidation), Q(Deamidation), S(Phosphorylation), T(Phosphorylation), Y(Phosphorylation)	fixed: C(Carbamidomethylation); variable: M(Oxidation)			
**Available Spectrums Count**	109038	125071	461761	596372			
**URL**	https://www.abrf.org/proteome-informatics-iprg-	http://dx.doi.org/10.6019/PXD008844	http://proteomecentral.proteomexchange.org/cgi/GetDataset?ID=PXD010559	http://dx.doi.org/10.6019/PXD008808	http://proteomecentral.proteomexchange.org/cgi/GetDataset?ID=PXD011246	http://proteomecentral.proteomexchange.org/cgi/GetDataset?ID=PXD012645	http://dx.doi.org/10.6019/PXD012979

For a fair comparison, we have used the high-resolution tandem mass spectrometry (MS/MS) datasets provided by PointNovo. PointNovo performed a database search with PEAKS X to identify ground truth peptide labels. The post-translational modifications (PTMs) settings are included in the Methods. The peptide-spectrum matches (PSMs) identified at 1% false discovery rate in each dataset are split into training, validation and test sets at a ratio of 8:1:1. It is worth noting that the merged dataset (including PXD008808, PXD011246, PXD012645 and PXD012979) does not have a test set due to the use of external datasets in the estimate of the generalizability, and it was finally split into training, validation sets at a ratio of 19:1. All of the above datasets are available in GitHub (https://github.com/shallFun4Learning/PGPointNovo).

Supplementary Table S3. Time-consuming against PointNovo

Model	Dataset	ABRF	PXD008844	PXD010559
PointNovo	Training Time (Hour)	7.52	8.03	86.55
	Inference Time(seconds)	255.61	213.61	1004.54
PGPointNovo (2GPUs)	Training Time (Hour)	3.89	4.21	44.01
	Inference Time(seconds)	133.99	108.18	550.65
	Training Speedup	1.93x	1.91x	1.97x
	Inference Speedup	1.90x	1.97x	1.82x
PGPointNovo (4GPUs)	Training Time (Hour)	2.11	2.23	22.64
	Inference Time(seconds)	69.83	56.97	280.33
	Training Speedup	3.56x	3.60x	3.82x
	Inference Speedup	3.65x	3.75x	3.58x
PGPointNovo (8GPUs)	Training Time (Hour)	1.04	1.09	11.83
	Inference Time(seconds)	36.25	30.02	142.01
	Training Speedup	7.23x	7.35x	7.32x
	Inference Speedup	7.05x	7.11x	7.07x

We compared with PointNovo on three benchmark datasets (More information on the dataset is in Supplementary Table S2), achieving a 1.91x – 7.35x training speedup and a 1.97x – 7.11x inference speedup against PointNovo. We can see that PGPointNovo significantly reduces the training time and inference time from scratch and obtains a near linear speed-up ratio which is essential for downstream tasks such as *de novo* peptide sequencing of new species.

Supplementary Table S4. Performance against PointNovo

Dataset	GPUs	AA Recall (%)	AA Precision (%)	Peptide Recall (%)
ABRF	Baseline	71.7	71.8	38.2
	PGPointNovo (2 GPUs)	71.8	71.9	38.3
	PGPointNovo (4 GPUs)	71.8	71.8	38.2
	PGPointNovo (8 GPUs)	71.7	71.9	38.3
PXD008844	Baseline	73.0	72.6	51.5
	PGPointNovo (2 GPUs)	73.0	72.7	51.8
	PGPointNovo (4 GPUs)	73.0	72.6	51.6
	PGPointNovo (8 GPUs)	73.0	72.7	51.6
PXD010559	Baseline	75.0	74.8	50.8
	PGPointNovo (2 GPUs)	75.1	74.9	51.2
	PGPointNovo (4 GPUs)	75.2	74.9	51.4
	PGPointNovo (8 GPUs)	75.3	75.0	51.4

This experiment was done with PointNovo's results as a baseline, under fair parameter settings (Supplementary Table S1). The result shows that PGPointNovo does not sacrifice identification precision or recall and shows even better performance than PointNovo on the larger PXD010559 dataset.

Supplementary Table S5. Ablation Test

Model	Learning Rate	AA Recall (%)	AA Precision (%)	Peptide Recall (%)
baseline	0.001	71.9	71.7	43.3
PGPointNovo	0.008	63.9	63.1	32.7
PGPointNovo with the Ranger	0.008	71.9	71.9	43.7
PGPointNovo	0.001	70.9	70.6	42.0
PGPointNovo with the Ranger	0.001	72.7	72.6	45.4

This experiment was trained on the merged dataset (merged by PXD008808, PXD011246, PXD012645 and PXD012979) and tested on an external dataset (merged the test set of ABRF, PXD008844 and PXD010559) using eight GPUs, again using the results from PointNovo (default learning rate of 0.001) as the baseline.

Although it is common practice to scale the learning rate with the number of GPUs in parallelisation, it is not always effective. At `lr=0.008`, PGPointNovo showed an error of more than 10% on the combined dataset. Considering the potential controversy associated with the learning rate, we tried not adjusting the initial learning rate (`lr=0.001`), but the error persisted. After activating the Ranger, performance improved and reached the baseline at two different learning rates, up to 0.8%, 0.9% and 2.1% for AA Recall, AA Precision and Peptide Recall respectively. The results demonstrate the generalizability of PGPointNovo and the availability of the Ranger.

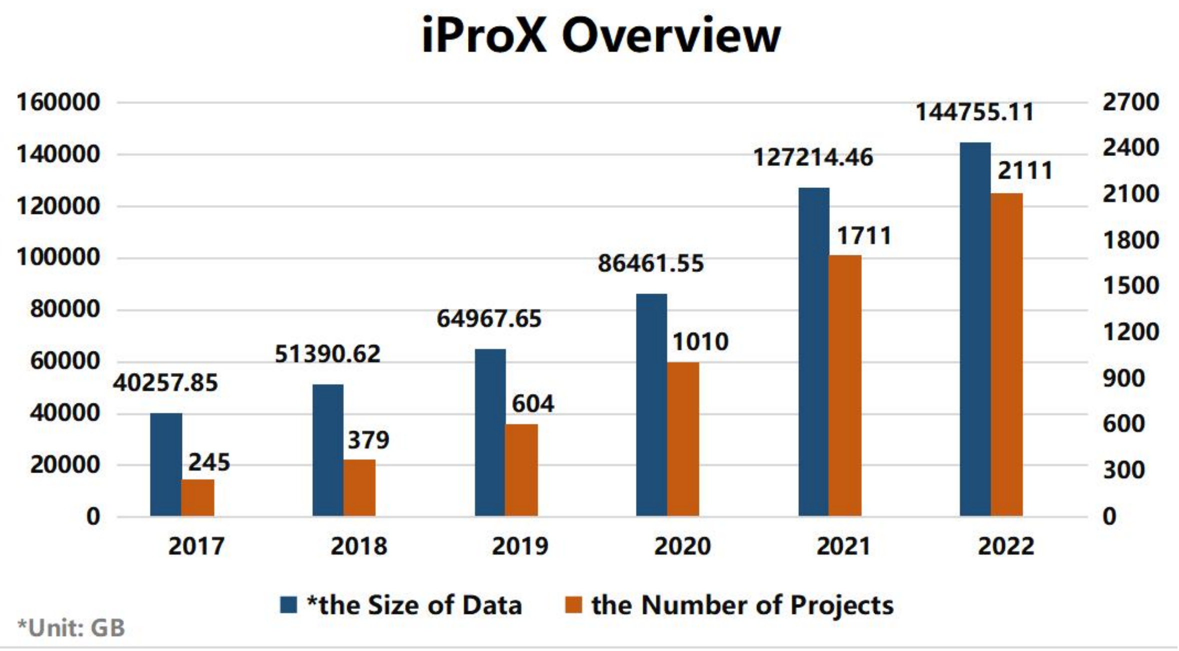

Supplementary Figs S1. iProX Overview

iProX is an integrated proteome resources center in China, which is built to accelerate the worldwide data sharing in proteomics(Chen, et al., 2022). As an official member of ProteomeXchange Consortium(Deutsch, et al., 2020), iProX assigns ProteomeXchange identifiers (PXD) to datasets submitted to iProX. Since its launch in 2017, the amount of data submitted to iProX has been increasing rapidly, reaching a total of 141 TB of data as of June 2022.

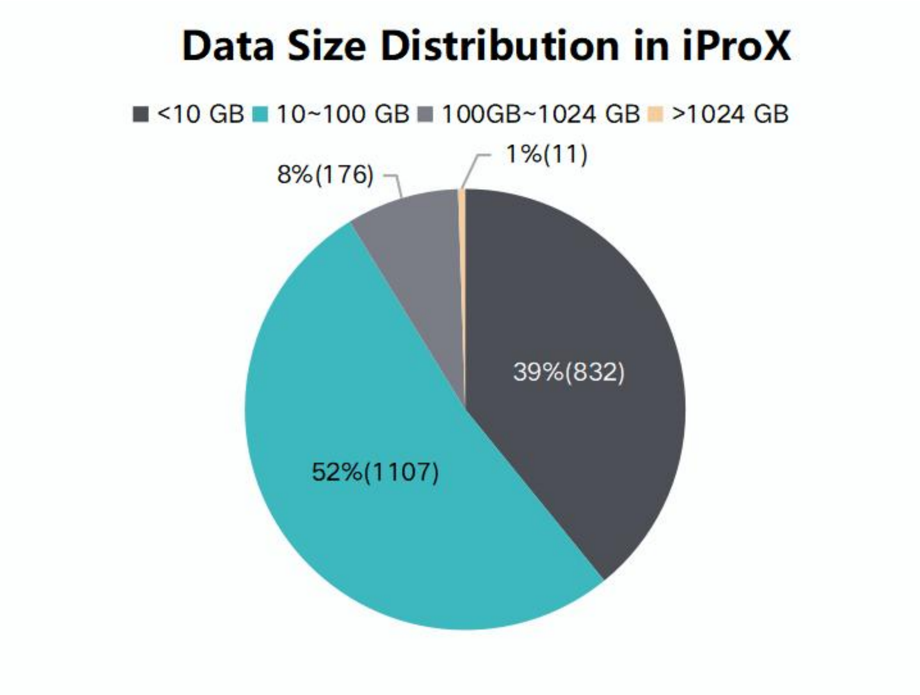

Supplementary Figs S2. Data Size Distribution in iProX

As of June 2022, there are over 2,000 data sets on iProX. More than 1,800 of these data sets are over 10GB, and 12 are over 1TB. PointNovo takes long time, sometime months, to process these data sets, which is undesirable, if not unacceptable, in practice.

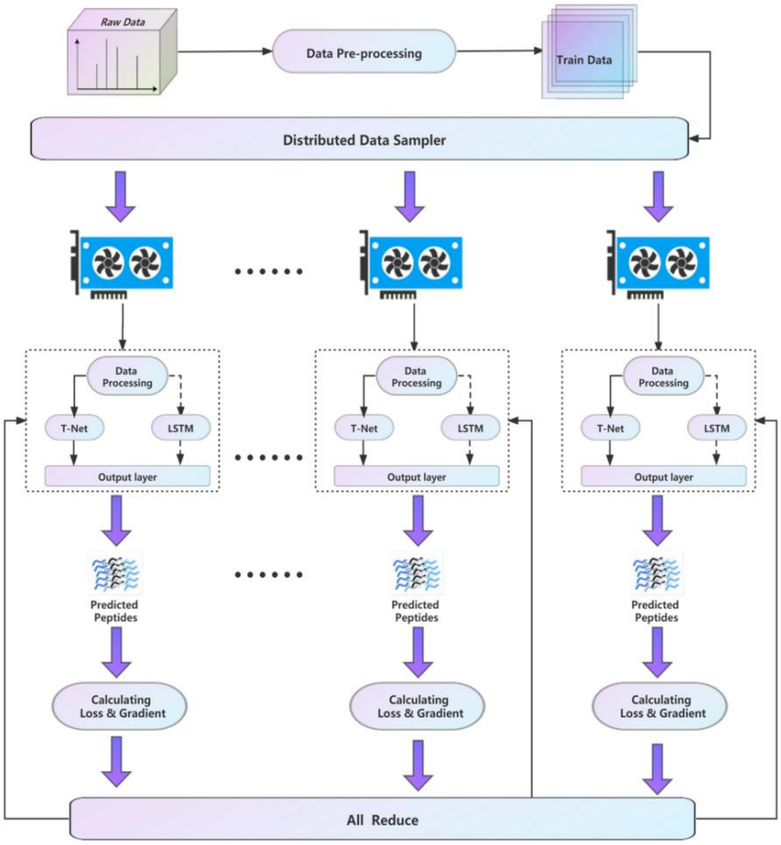

Supplementary Figs S3. PGPointNovo Parallelisation Framework

PGPointNovo first pre-processes raw spectral data into a conformed format and then distributes formatted data to individual GPUs for training. Each GPU trains a complete neural network model independently with identical initialization parameters. Data consistency and diversity are guaranteed by the Distributed Data Sampler during the data distribution process. Each GPU receives a copy of the data and processes it in a single card process and calculates losses and gradients. Before the parameters are finally updated, the corresponding gradients are broadcasted via an all-reduce operation. This process is does not involve user intervention. The user only needs to focus on the model, which enables easy use of PGPointNovo in practice.

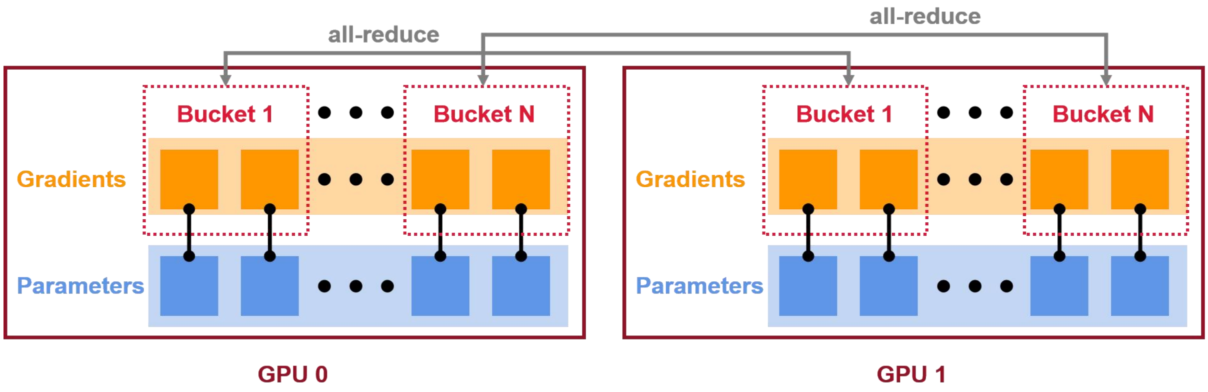

Supplementary Figs S4. all-reduce

PyTorch autograd engine accepts custom backward hooks (PyTorch, 2022). It allows us to employ autograd hooks to enable gradient synchronization. When the target gradient is generated, an autograd hook corresponding to the target gradient will be triggered and marked with a special mark. For communication efficiency reasons, gradients are mapped into buckets. When all the gradients in the same bucket are ready (by checking the flags from autograd hooks), the all-reduce operations between buckets will be started. When these are completed, every GPU will obtain the same average gradient.

Part2. Availability, Installation and Usage of PGPointNovo

PGPointNovo is written Python. The use of PGPointNovo requires basic understanding of Python.

Availability

PGPointNovo is available at (https://github.com/shallFun4Learning/PGPointNovo).

Installation and Requirements

python >= 3.7.1

pytorch >= 1.7.1

dataclasses

biopython,

pyteomics,

cython

Usage

PGPointNovo employs a similar set of commands to PointNovo's, which ensures a smooth transition for users from PointNovo to PGPointNovo. Its new optimisation strategy and important parameters can be switched on/off by editing the `config.py` file. PointNovo(Qiao, et al., 2021) users can easily experiment PGPointNov with a variety of parameters.

For new users, PGPointNovo can be launched with a simple command `make run`. It will automatically configure the relevant training files and setting information, such as the number of nodes in the `makefile` file and runs PGPointNovo.

Due to the limitations of the doc format, more detailed usage will be available on GitHub(https://github.com/shallFun4Learning/PGPointNovo/blob/main/README.md), feel free to submit a new issue if you encounter any problems.

Part3. Conclusion and forecast of the research

With the rapid increase in mass spectrum data, it is a grand challenge to sequence a huge volume of data efficiently. This paper presents PGPointNovo, an efficient neural network-based tool for parallel *de novo* peptide sequencing with data parallelism. Extensive experiments conducted on multiple datasets of different sizes demonstrate that PGPointNovo achieves profound speedups against the excellent ap-proach without sacrificing precision, recall and generalizability. In the future, we will attempt accelerate *de novo* peptide sequencing in GPU clusters, and investigate the application data parallelism to other *de novo* applications.

REFERENCES

Chen, T., *et al.* iProX in 2021: connecting proteomics data sharing with big data. *Nucleic Acids Res* 2022;50(D1):D1522-D1527.

Deutsch, E.W., *et al.* The ProteomeXchange consortium in 2020: enabling 'big data'approaches in proteomics. *Nucleic acids research* 2020;48(D1):D1145-D1152.

PyTorch. Extending PyTorch. In.; 2022.

Qiao, R., *et al.* Computationally instrument-resolution-independent *de novo* peptide sequencing for high-resolution devices. *Nature Machine Intelligence* 2021;3(5):420-425.
